# Genetic Diversity of *Cryptosporidium* in Children in an Urban Informal Settlement of Nairobi, Kenya

**DOI:** 10.1371/journal.pone.0142055

**Published:** 2015-12-21

**Authors:** Cecilia Mbae, Erastus Mulinge, Anthony Waruru, Benjamin Ngugi, James Wainaina, Samuel Kariuki

**Affiliations:** 1 Centre for Microbiological Research, Kenya Medical Research Institute, Nairobi, Kenya; 2 Kenya Medical Research Institute, Nairobi, Kenya; 3 Bioscience eastern and central Africa, International Livestock Research Institute, Nairobi, Kenya; Instituto de Higiene e Medicina Tropical, PORTUGAL

## Abstract

**Introduction:**

Globally *Cryptosporidium* and *Giardia* species are the most common non-bacterial causes of diarrhoea in children and HIV infected individuals, yet data on their role in paediatric diarrhoea in Kenya remains scant. This study investigated the occurrence of *Cryptosporidium* species, genotypes and subtypes in children, both hospitalized and living in an informal settlement in Nairobi.

**Methods:**

This was a prospective cross-sectional study in which faecal specimen positive for *Cryptosporidium spp*. by microscopy from HIV infected and uninfected children aged five years and below presenting with diarrhoea at selected outpatient clinics in Mukuru informal settlements, or admitted to the paediatric ward at the Mbagathi District Hospital were characterized. The analysis was done by Polymerase Chain Reaction-Restriction Fragment Length Polymorphism (PCR-RFLP) of the 18srRNA gene for species identification and PCR-sequencing of the 60 kDa glycoprotein *(GP60)* gene for subtyping.

**Results:**

*C*. *hominis* was the most common species of *Cryptosporidium* identified in125/151(82.8%) of the children. Other species identified were *C*. *parvum* 18/151(11.9%), while *C*. *felis* and *C*. *meleagridis* were identified in 4 and 2 children, respectively. Wide genetic variation was observed within *C*. *hominis*, with identification of 5 subtype families; Ia, Ib, Id, Ie and If and 21 subtypes. Only subtype family IIc was identified within *C*. *parvum*. There was no association between species and HIV status or patient type.

**Conclusion:**

*C*. *hominis* is the most common species associated with diarrhoea in the study population. There was high genetic variability in the *C*. *hominis* isolates with 22 different subtypes identified, whereas genetic diversity was low within *C*. *parvum* with only one subtype family IIc identified.

## Introduction

The genus *Cryptosporidium* is a multispecies complex with extensive genetic variation. Approximately 27 species and more than 60 genotypes have been identified [1–4]. A wide diversity of *Cryptosporidium spp*. and subtypes infect humans, and each may have a range of transmission routes of public-health significance [5,6]. DNA analysis of *Cryptosporidium* parasites from humans has shown the anthroponotic *C*. *hominis* and the zoonotic *C*. *parvum* to be the most common cause of human cryptosporidial infections, with 90% of reported cases attributed to them[5,6]. Recently an additional 8 species have been identified as causes of cryptosporidiosis in humans, including *C*. *meleagridis*, *C*. *felis*, *C*. *canis*, *C*. *suis*, *C*. *muris*, *C*. *fayeri*, *C*.*ubiquitum*, and *C*. *cuniculus* [7–10]. The contribution of these species to human cryptosporidiosis varies globally and has been documented to be associated with seasonality, demographics, immune status, and contact with reservoir hosts [1].

Further intra-species variation has been observed in *Cryptosporidium* isolates which further classifies species to the subtype family and subtype levels. To date six *C*. *hominis* subtype families (Roman numeral I) and 11 *C*. *parvum* subtype families (Roman numeral II) [1] have been identified. Six subtype families have also been identified in *C*. *meleagridis* (Roman numeral III) and *C*. *fayeri* (Roman numeral IV) [11,12].

Advances in molecular biology and full genome sequencing has contributed to knowledge on molecular epidemiology and better understanding of the biology and transmission of cryptosporidiosis in humans, as well as associations of different species or subtypes to clinical manifestations and infection risk factors, such as age and HIV status [1,13]. In addition, information gained from such studies provides support for treatment, prevention and control strategies [14].

Amplification and sequencing of one or more genetic loci (markers) have been used for the differentiation of *Cryptosporidium* species, genotypes and subtypes [11,15]. In particular, a PCR-RFLP tool that targets ~830-bp fragment of the small subunit (SSU) rRNA gene and uses *SspI* and *VspI* restriction enzymes for genotyping [16,17] is commonly used, due to the multi-copy nature of the gene and presence of semi-conserved and hyper-variable regions. Other genotyping tools based on the oocyst wall protein (COWP) gene have narrow specificity and only amplify DNA of *C*. *parvum*, *C*. *hominis*, *C*. *meleagridis*, and species/genotypes closely related to *C*. *parvum* hence this technique is rarely used for diverse samples[1,18].

Mini- and micro-satellites, or simple sequence repeats, constitute a rich source of polymorphism and have been extensively used for high-resolution genotyping and subtyping [19]. In particular, the *GP60* gene is useful for such studies as it contains multiple regions with high mutation rates, including a ‘hyper-variable’ microsatellite region [20]. The *GP60* gene is the most polymorphic marker identified so far in the *Cryptosporidium* genome and exhibits extensive sequence differences in the non-repeat regions, which categorize *C*. *parvum* and *C*. *hominis* each to several subtype families [21]. Within each subtype family, differences are attributed to the number of trinucleotide repeats (TCA, TCG or TCT microsatellite) [22,23]. Molecular analysis of the *GP60* gene has facilitated the identification of transmission pathways and zoonotic disease contamination sources and highlighted the importance of certain genetic variants to human health, and the public health risk posed by particular *Cryptosporidium* subtypes. There are also significant differences in clinical presentations and virulence among some common *C*. *hominis* subtype families in cryptosporidiosis- endemic areas [13,24]. Although cryptosporidiosis is prevalent in Sub Saharan Africa, information on the molecular characteristics of *Cryptosporidium spp*. is scant. In Kenya particularly, *Cryptosporidium* has been found to be a major cause of diarrhoea in children [25,26], yet information on genetic diversity of circulating *Cryptosporidium* spp. and subtypes in different populations is limited.

The aim of this study was to determine the prevalence, epidemiology and the genetic diversity of *Cryptosporidium spp*. isolated from children with diarrhoea, presenting at outpatient clinics in Mukuru informal settlement, or admitted at the Mbagathi District Hospital. The understanding of this relationship may represent the starting point for further extended studies on the epidemiology and genetic diversity of *Cryptosporidium* in different populations, and establish routes of transmission and intervention measures.

## Materials and Methods

### DNA isolation

Genomic DNA was extracted from faecal specimens that were positive for *Cryptosporidium* from an earlier published study (Mbae *et al*., 2013)using QiAmp^®^ DNA stool Mini kit (Qiagen, Crawley, West Sussex, United Kingdom) with slight modifications. Briefly 200 μl of fecal suspension was washed five times with triple-distilled water by centrifugation. To this suspension 1.4 ml of ASL buffer was added and subjected to five times thawing (80°C) and freezing (-80°C) to rupture the rigid oocysts. The genomic DNA was eluted in 50 μl of Nuclease free water and stored at -20°C until use.

### Species and genotype identification

PCR Amplification targeting the *Cryptosporidium* 18S rRNA and restriction fragment length polymorphism (RFLP) of the PCR amplicon were used to genotype *Cryptosporidium* isolates essentially as described [27]. Briefly, a two stage nested PCR of the 18S rRNA gene was done; the primary PCR amplified a 1,325 bp, using forward primer 5’-TTCTAGAGCTAATACATGCG-3’ and reverse primer 5’-CCCATTTCCTTCGAAACAGGA-3’. The illustra^™^ PuReTaq Ready-To-Go PCR Beads (GE Healthcare, UK) was used for amplification in a 25 μl final volume, to the beads (2.5 units of puRe Taq DNA polymerase, 200 μM of each dNTP, 10 mM Tris-HCl pH 9.0, 50 mM KCl, 1.5 mM MgCl_2_), 0.25 μM of each primer, an extra 1.5 mM MgCl_2_, 21.25 μl of nuclease free water and 1.0 μl of DNA were added. The secondary amplification of an internal fragment of between 826–864 bp was done using forward primer5’-GGAAGGGTTGTATTTATTAGATAAAG-3’and reverse primer 5’-CTCATAAGGTGCTGAAGGAGTA-3’. The same conditions described for primary PCR were used for the secondary PCR reaction except 0.5 μM of each primer, 20 μl of nuclease free water and 1 μl of the primary PCR product were added to the beads. Two step restriction digestion of the secondary PCR productswas carried in a total reaction volume of 40 μl. The reaction mix composed of; 15 μl of the secondary PCR amplicon as the template, 2 μl (20 units) of *SspI* (Promega) and 4 μl of 1 X restriction buffer. The second set of restriction digestion using *VspI* (Promega) enzyme was also performed in a total reaction volume of 40 μl, consisting of 1 μl (10–12 units) of the enzyme, with similar volumes of buffer and PCR amplicons as the *SspI* reaction. Digestion was carried out in a water bath at 37°C for a minimum of 4 hours. The digestion products were resolved on 2% agarose gel stained with ethidium bromide and visualized under UV. Fragment sizes were then compared with the expected banding patterns from known *Cryptosporidium* species and genotypes as previously published [28] for species/genotype identification. DNA sequencing was carried out only on isolates that did not give distinct bands for confirmation.

### C. parvum and C. hominis subtyping

Subtyping of *C*. *parvum* and*C*. *hominis* was carried out by a *GP60*-based tool, which amplified an~850 bp fragment of the *GP60* gene, and the sequences obtained analysed for identification of subtype families and subtypes, as described previously [28]. In the primary PCR forward primer (AL3531 5’-ATAGTCTCCGCTGTATTC-3’) and reverse primer (AL3535 5’-GAGATATATCTTGGTGCG-3’) were used, while the forward primer (AL3532 5’-TCCGCTGTATTCTCAGCC-3’) and reverse primer (AL3534 5’-GCAGAGGAACCAGCATC-3’) were used for the nested PCR. The same conditions described for 18rRNA PCR were used for *GP60* semi-nested PCR. Reaction mixtures containing the correct size fragment were purified using QIAquick PCR purification kit (Qiagen, Crawley, West Sussex, United Kingdom) according to the manufacturer's protocol.

### Sequencing of GP60 PCR products

The purified nested PCR amplicons of *Cryptosporidium* isolates of *GP60* gene, were sequenced in both directions using forward primer AL3532 and reverse primer AL3534[29]. Bidirectional sequencing of DNA samples using ABI3730 and Big Dye terminator v3.1 kit was carried out at the International Livestock Research Institute, BeCA laboratories, Nairobi, Kenya. Base calling for each sequence run was done using Sequence Analysis v5.2 software Sequences received were first edited and consensus sequence generated from the forward and reverse sequence using CLC DNA workbench 6.1. Each consensus sequence from individual isolates was used for the identification of *Cryptosporidium GP60* subtypes. Basic local alignment search tool (BLAST) (www.ncbi.nlm.nih.gov/blast) was used to assess identity and degrees of similarities with *CryptosporidiumGP60* subtypes in the GenBank as well as compared with other reference sequences of *C*. *parvum* and *C*. *hominis* subtypes from different geographical regions.

Multiple sequence alignment of *Cryptosporidium* isolates with GenBank reference sequences of *C*. *parvum* and *C*. *hominis* subtypes were aligned using ClustalX 2.1 [29]. The phylogenetic analysis of the *GP60* gene nucleotide sequence data were conducted using the software package MEGA vs.5.05 [30]. The evolutionary relationship was inferred using the Neighbor-Joining method. Branches that had less than 50% bootstrap value were collapsed. The tree was rooted using *C*. *meleagridis* (Accession No. AF401499) as the outgroup.

The previously established nomenclature system was used to differentiate subtype families and subtypes within each species [22]. All the *GP60Cryptosporidium* sequences were analysed for ‘‘TCA” microsatellite region. *GP60* sub genotype results analysis display high mutation rates, in particular, a ‘‘hyper-variable” microsatellite region. The *GP60* sub-genotype ‘‘TCA” micro satellite region, showed triplet cordons were categorized according to the number of trinucleotide repeats coding for the amino acid serine. *Cryptosporidium GP60*subtypes consist of a variable number of ‘‘A” (TCA), ‘‘G” (TCG), ‘‘T” (TCT) and ‘‘R” (ACATCA) [1,22]. The PCR-RFLP of the *18S rRNA* gene and sequence analysis of the *GP60*gene locus provided information on the genetic inter-relationships of the C*ryptosporidium spp*. in the study population.

### Statistical analysis

Data from the questionnaires was entered into a Microsoft access database using EpiInfo^™^ version 3.3 (CDC, 2004). Data cleaning procedures were performed before importing data for analysis into Stata 9.2 (Stata Corporation, Texas USA) for analysis. Frequencies and proportions for patients’ characteristics categorized by cryptosporidium species, families and subtypes were calculated and reported.

Univariate analyses were used to identify potential patients’ characteristics, clinical symptoms and seasonality correlates of infection with particular cryptosporidium species and assessed for significance to determine suitability for multivariate logistic regression analyses. Odds ratios were used to describe associations and a p-value of <0.05 was considered significant.

### Ethical approval

The study was approved by the Kenya National Ethical Review Committee. All guardians of participating children were informed of the study objectives and voluntary consent was sought before inclusion.

## Results

### Genotyping Cryptosporidium from faecal samples

**Fig 1:**
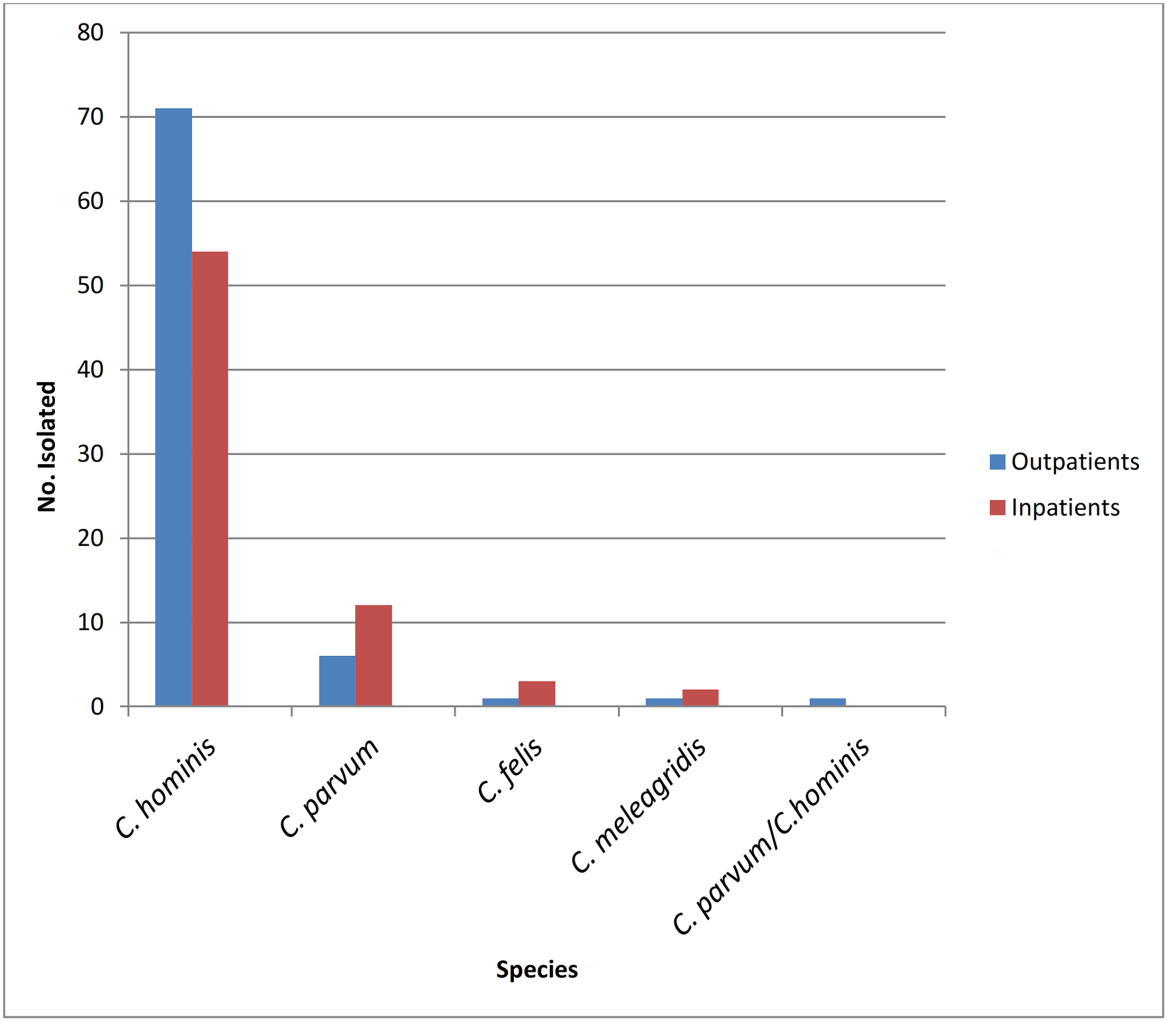
Distribution of *Cryptosporidium* species. Distribution of *Cryptosporidium* species **among children at the outpatient clinics in Mukuru and those admitted in paediatric ward at Mbagathi District Hospital**.

A total of 151 out of 187 samples positive by microscopy from a previously published study [25]. were genotyped. Thirty six samples that were positive by microscopy did not give an amplication by 18S rRNA PCR. Results from PCR-RFLP analysis of 18S rRNA PCR revealed banding patterns distinctive of four different species. Majority of the isolates;125/151(82.78%),were identified as *C*. *hominis*, making it the most predominant species identified in the study population. Among these, 71(56.8%) were outpatients and 54 (43.2%) inpatients. *C*. *parvum* was identified in 18/151(11.92%), 6 (33.3%) of which were from outpatients and 12 (66.67%) from inpatients. Among the other samples, 4/151(2.64%) were identified as *C*. *felis*. Three (75%) of *C*. *felis* positive samples were isolated from inpatients and 1(25%) from outpatients. Lastly, *C*. *meleagridis* was seen in 2/151(1.32%) of the samples analysed, and these came from children recruited at the outpatient clinics. Mixed infection with *C*. *parvum* and *C*. *hominis* was seen from an outpatient. Occurrence of these species in relation to patient type (inpatients and outpatients) is shown in Fig 1.

### Sub-typing C. parvum and C. hominis using GP60 based tool

A total of 101 isolates were successfully amplified using the *GP60* gene target, and the nested PCR amplicons sequenced. The remaining samples did not give amplification with the *GP60* PCR, while one sample that had not amplified with 18S rRNA PCR amplified with *GP60* PCR. Of these, 82/101 (81.2%) were identified as *C*. *hominis*, while 19/101 (18.8%) were *C*. *parvum*.

Age-specific distribution of cryptosporidiosis due to the two species showed the highest prevalence in the 0–24 months -old age group, with all children except one (18/19, 94.8%), infected with *C*. *parvum* belonging to this age-group. The odd one was isolated in an older child in the 49–60 months-old age group. With *C*. *hominis*, highest prevalence was observed in 0–12 months age group (45.7%), followed by 13–24 months age-group (33.3%), while 13.6% were isolated in 25–36 month age-group. Only 3 isolates were from those over 36 months old (Table 1).

**Table 1:** Distribution of *C*. *hominis* and *C*. *parvum* in relation to patient characteristics, clinical symptoms and seasonality.

		*C*. *hominis*				*C*. *parvum*			
Patient characteristics	N	n (%)	OR	95%CI	p-value	n (%)	OR	95%CI	p-value
Total	101	82(81.2)	-			19(18.8)	-		
Patient type									
Out-patient(ref)	51	44(86.3)	1			7(13.7)	1		
In-patient	50	38(76)	0.19	0.18,1.41	0.191	12(24)	1.98	0.71,5.55	0.191
Age group									
0 to 12 months(ref)	46	37(80.4)	1			9(19.6)	1		
13 to 24 months	36	27(75)	0.56	0.26,2.08	0.556	9(25)	1.37	0.48,3.91	0.556
25 to 36 months	11	11(100)	-			0(0)	-		
37 to 48 months	3	3(100)	-			0(0)	-		
49 to 60 months	4	3(75)	0.80	0.07,7.87	0.795	1(25)	1.37	0.13,14.77	0.795
HIV status									
Positive(ref)	34	25(73.5)	1			9(26.5)	1		
Negative	63	54(85.7)	2.16	0.76,6.10	0.146	9(14.3)	0.46	0.16,1.31	0.146
Acute diarrhoea									
No(ref)	44	36(81.8)	1			8(18.2)	1		
Yes	57	46(80.7)	0.93	0.34,2.55	0.887	11(19.3)	1.08	0.39,2.95	0.887
Chronic diarrhoea									
No(ref)	67	53(79.1)	1			14(20.9)	1		
Yes	34	29(85.3)	1.53	0.50,4.68	0.454	5(14.7)	0.65	0.21,1.99	0.454
Vomiting									
No(ref)	19	13(68.4)	1			6(31.6)	1		
Yes	82	69(84.1)	2.44	0.79,7.62	0.122	13(15.9)	0.41	0.13,1.27	0.122
Abdominal pain									
No(ref)	40	31(77.5)	1			9(22.5)	1		
Yes	61	51(83.6)	1.48	0.54,4.05	0.444	10(16.4)	0.68	0.25,1.85	0.444
Fever									
No(ref)	32	23(71.9)	1			9(28.1)	1		
Yes	69	59(85.5)	2.31	0.83,6.41	0.108	10(14.5)	0.43	0.16,1.20	0.108
Seasonality									
Dry season(ref)	25	20(80)	1			5(20)	1		
Wet season	76	62(81.6)	1.12	0.35,3.46	0.861	14(18.4)	0.90	0.28,2.82	0.861

There were more patients infected with *C*. *parvum* among the inpatients (63.2%), while equal numbers were infected with *C*. *parvum* among HIV infected and HIV uninfected children, with 9 cases in each group. Prevalence of *C*. *hominis* was 53.7% and 46.3% among outpatients and inpatients, respectively, while more HIV negative children were infected with *C*. *hominis* as compared with HIV positive children (68.4% vs. 31.6%) respectively. However the difference in these groups was not significant (Table 1).

Associated clinical manifestations varied between the different *Cryptosporidium spp*. Generally, *C*. *hominis* infections were associated with more diverse and severe clinical manifestations of fever, vomiting, abdominal pains and acute diarrhoea. Vomiting was observed in 84% of those infected with *C*. *hominis* and 68.4% of *C*. *parvum* infections, while fever was reported among the 72% of *C*. *hominis* and 53% of *C*. *parvum* infections. More children infected with *C*. *hominis* presented with chronic diarrhoea than those infected with *C*. *parvum*: 35% versus 26%, respectively. More cases of cryptosporidiosis due to *C*. *hominis* were identified during the wet season compared to dry season (80% *vs*.81.6%), and the same pattern was observed with *C*. *parvum* infections. These differences were not however significant (73.4% *vs*. 26%) (Table 1).

### Microsatellite analysis of *CryptosporidiumGP60* subtypes and subtype families

Phylogenetic relationships based on*GP60* nucleotides of the *C*. *hominis* sequences clustered isolates from this study into five distinct subtype families and one subtype family of *C*. *parvum* (Table 2). The *C*. *hominis* subtype families identified among 82 isolates were; Ia, (n = 5, 6.1%), Ib, (n = 20, 24.4%), Id, (n = 31, 37.8%), 1e, (n = 23, 28%) and If (n = 3, 3.7%). Among subtype family Ia, four subtypes were found: IaA7R1 (in two cases), IaA25R5 (in one case), IaA27R3 (in one case) and IaA30R3 (in one case). Within subtype family Ib, only two subtypes were identified with subtype IbA9G3 being the most common, present in 17 cases and 1bA9G3R2 which was present in 3 cases. Subtype family Id showed the highest diversity of *C*. *hominis* with 10 subtypes (S1 Table). These included IdA22, being the most common in 14 cases, IdA25 in 5 cases, IdA24 and IdA15G1 in 3 cases each while subtypes IdA19, IdA21, IdA20, IdA18, IdA17G1 and IdA23G1 were identified in one case each. Subtype family Ie was the least diverse with subtype Ie11G3T3R1 in 19 samples, while 4 samples belonged to subtype IeA11G3T3. Subtype family If had 3 subtypes, comprising IfA19G1, IfA14G1, IfA12G1and these were identified in one case each (Table 2).

**Table 2:** *CryptosporidiumGP60* subtype families and subtypes identified in 101 samples from children with diarrhoea from selected outpatient clinics in Mukuru informal settlement and paediatric ward in Mbagathi District Hospital, Nairobi, Kenya.

	*GP60* Subtype family	*GP60* Subtype	No. of isolates
***C*. *hominis***	**Ia**	IaA25R5	1
		IaA27R3	1
		IaA30R3	1
		IaA7R1	2
	**Ib**	IbA9G3	17
		IbA9G3R2	3
	**Id**	IdA22	14
		1dA24	3
		IdA19	1
		IdA25	5
		IdA21	1
		IdA20	1
		IdA17G1	1
		IdA18	1
		IdA15G1	3
		IdA23GI	1
	**Ie**	IeA11G3T3R1	19
		IeA11G3T3	4
	**If**	IfA19G1	1
		IfA14G1	1
		IfA12G1	1
**Subtotal**	**5**	**21**	**82**
***C*.*parvum***	**IIc**	IIcA5G3R2	19
**Subtotal**	**1**	**1**	**19**
**Total**	**6**	**22**	**101**

Nucleotide sequence of *GP60* in *C*. *parvum* recognized the presence of a single subtype family, IIc from positive patients and all belonged to subtype IIcA5G3R2.

*C*. *hominis* distribution across age groups was indiscriminate, with subtype families Ia, Ib, Id and Ie more frequently isolated in younger children than older ones. Isolation of the three cases of subtype If were confined to children within age group 0–12 months- old category. Subtype family Ia was isolated from HIV positive outpatients with a single isolate from HIV negative patient. Subtypes IA25R5, IaA27R3 and IaA27R1 were all from HIV negative outpatients, while IaA30R3 was identified in an outpatient HIV infected child. The most common subtype identified, IbA9G3, was present in four HIV infected outpatients and two HIV infected inpatients, with the rest being among HIV negative children. Two of the subtypes IbA9G3R2 were detected in HIV positive inpatients and 1 in HIV negative outpatient.

The most common subtype within subtype family Id, i.e. IdA22 was identified in 6 HIV negative inpatients, 4 HIV negative outpatients, 3 HIV positive inpatients and 1 HIV positive outpatient. Almost equal numbers were infected with subtype family Ie at the outpatient and inpatient settings (11cases and 12 cases respectively). Two out of the three subtype family If cases were in inpatients who were HIV negative and one was from an HIV positive outpatient Detailed distribution of subtype families isolated based on HIV status, patient type, age and other factors is shown in Table 3.

**Table 3:** Distribution of *GP60* subtype families in relation to patient characteristics, presenting clinical symptoms and other factors. Distribution of *C*.*hominis* and *C*. *parvum GP60* subtypes in relation to gender, HIV status, patient type, seasons and clinical symptoms is shown.

Patient characteristics	Total	*C*. *hominis GP60 Subtype families*					*C*. *parvum GP60 subtype*
		Ia	Ib	Id	Ie	If	IIc
**All patients**	101	5	20	31	23	3	19
**Gender**							
**Male**	55	3	12	14	15	1	10
**Female**	46	2	8	17	8	2	9
**Age group**							
**0 to 12 mos**	46	2	5	17	10	3	9
**13 to 24 mos**	36	0	7	11	9	0	9
**25 to 36 mos**	11	1	4	2	4	0	0
**37 to 48 mos**	3	1	1	1	0	0	0
**49 to 60 mos**	4	0	3	0	0	0	1
**HIV status**							
**Positive**	34	1	8	10	5	1	9
**Negative**	63	4	12	19	17	2	9
**Patient type**							
**Outpatient**	51	5	12	15	11	1	7
**Inpatient**	50	0	8	16	12	2	12
**Wet/dry season**							
**Dry**	25	2	3	10	5	0	5
**Wet**	76	3	17	21	17	3	14
**Acute diarrhoea**							
**No**	44	5	9	14	8	0	8
**Yes**	57	0	11	17	15	3	11
**Chronic diarrhoea**							
**No**	67	1	13	19	17	3	14
**Yes**	34	4	7	12	6	0	5
**Vomiting**							
**No**	19	2	1	5	5	0	6
**Yes**	82	3	19	26	18	3	13
**Abdominal pain**							
**No**	40	1	5	10	14	1	9
**Yes**	61	4	15	21	9	2	10
**Fever**							
**No**	32	2	2	9	9	1	9
**Yes**	69	3	18	22	14	2	10

Acute diarrhoea, vomiting, abdominal pain and fever was recorded more in children with subtype family Id, the most commonly identified *C*. *hominis* subtype, than other subtype families.

Patterns of clinical manifestations also varied among *C*. *hominis* subtype families. All the 5 subtype Ia infections were associated with chronic diarrhoea with none associated with acute diarrhoea, while all the other subtypes were associated with acute diarrhoea. Notably 3 subtype If cases were associated acute diarrhoea. All the subtype families except Ie, showed similar patterns of distribution with vomiting, abdominal pains and fever (Table 3).

### Phylogenetic relationships of C. hominis and C. parvumbased on GP60 subtyping tool

**Fig 2:**
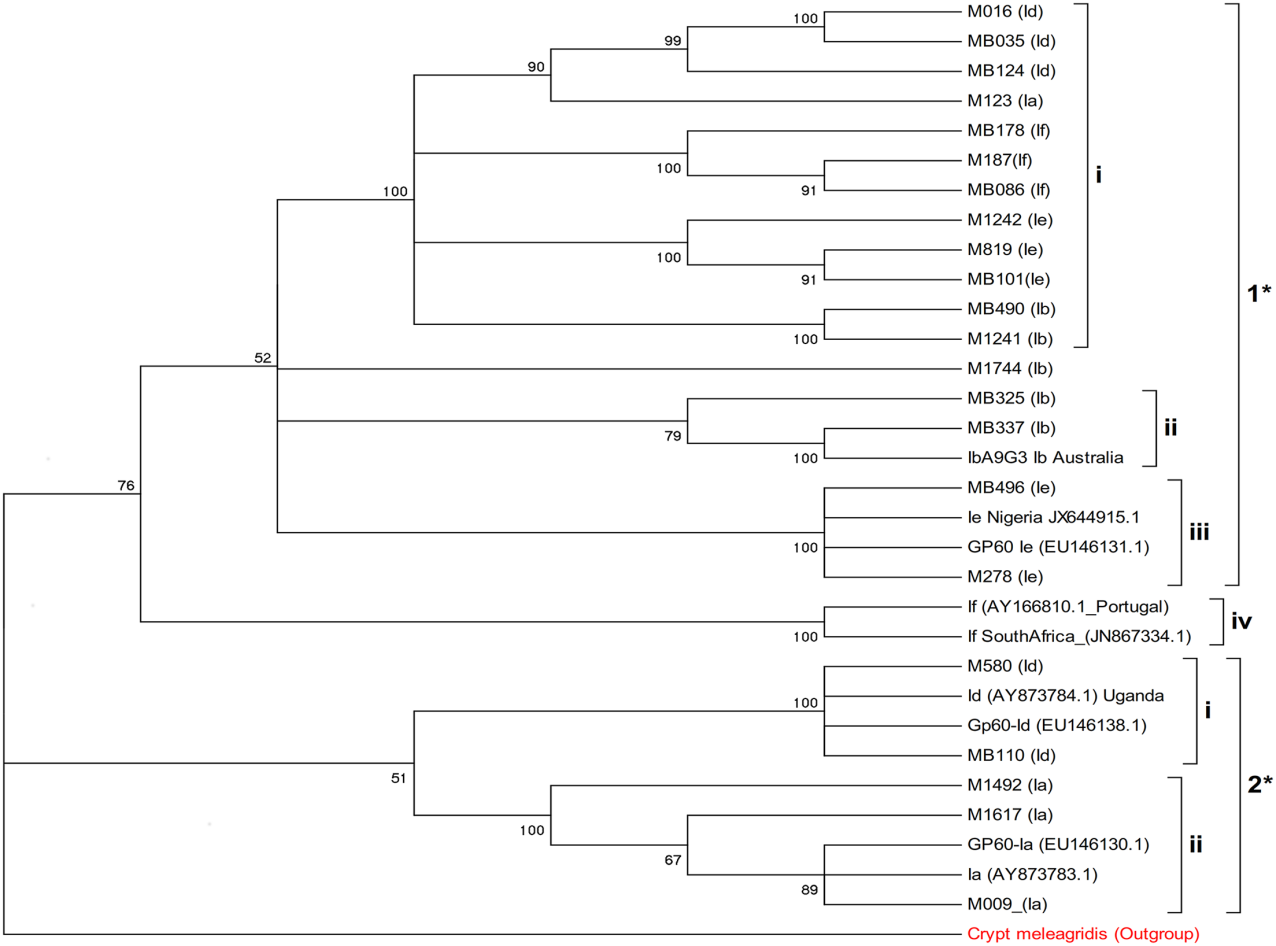
Phylogenetic relationship of selected *C*. *hominis* subtypes isolated from patients. The evolutionary history was inferred using the Neighbor-Joining method [31]. The bootstrap consensus tree inferred from 2000 replicates is taken to represent the evolutionary history of the taxa analyzed [32]. The samples are coded according to where they were recruited from and patient number. M187 refers to Mukuru patient (outpatient) number 187, MB110 refers to Mbagathi patient (inpatient) number 110. The subtype family is indicated in brackets.

Phylogenetic analysis of the *GP60* gene, from selected *Cryptosporidium* isolates showed two distinct clades; clade 1 and 2 (Fig 2). Clade 1, was composed of, four main sub-clades i –iv. Sub-clade i was composed of an admixture of subtype (Ib, If, Ie, Ia, Id), from Kenyan isolates. Sub-clade ii, contained the subtype Ib, with Kenya isolates clustering very closely with isolates from Australia. The sub-clade III, was composed of subtype Ie with the Kenyan isolates comparable with Nigeria isolates from retrieved from GenBank. Sub-clade IV was composed of isolates from the subtype If, that clustered with South Africa isolates retrieved from the GenBank. The second major clade 2, was composed of two sub-clades, i and ii, that were contained subtype families Id and Ia respectively (Fig 2). Multiple sequence alignment of *Cryptosporidium* subtype Id against references Genbank sequences (S1 Fig), indicated single point mutations/substitution within the sequences.

**Fig 3:**
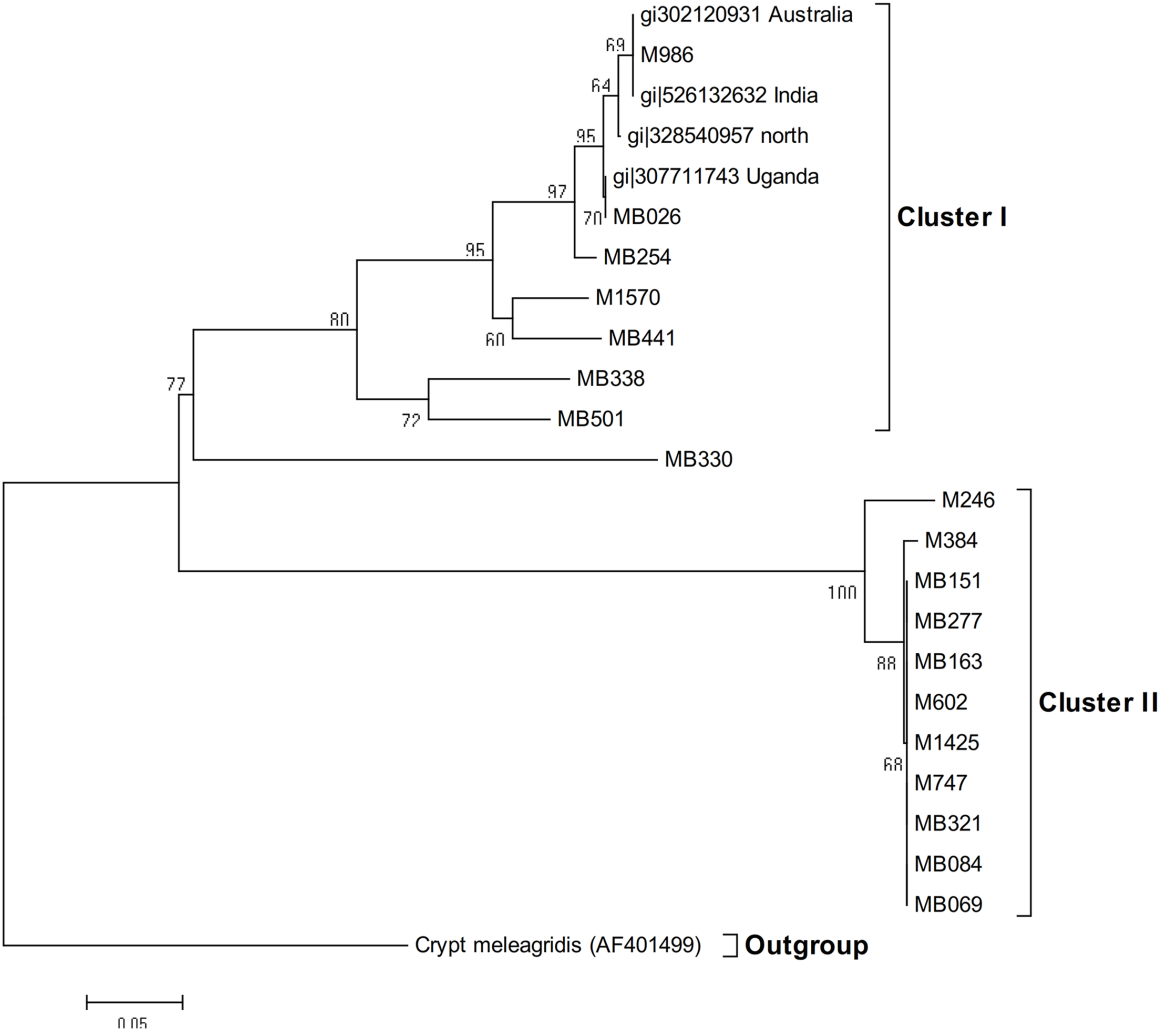
Phylogenetic relationship of selected *C*. *parvum* isolated from patients. Evolutionary analyses were conducted in MEGA5 [33]. The samples are coded according to where they were recruited from and patient number. M602 refers to Mukuru patient (outpatient) number 602, MB336 refers to Mbagathi patient (inpatient) number 336.

Representative phylogenetic relationship within the *C*. *parvum* isolates had two distinct clades (Fig 3). Clade I was composed mainly of Kenyan isolates, that clustered with bootstrap values of >50% with reference isolates from Australia and India and Uganda, with Clade II composed of distinct *C*. *parvum* isolates from Kenya, that did not cluster with any of the reference samples from the Genbank. The accession numbers of the reference sequences are indicated on the dendrograms.

## Discussion

The present study produced data on the importance of *Cryptosporidium spp*., and the predominant species and subtypes circulating in this patient population of children presenting with diarrhoea in an informal settlement of Nairobi. The results also show the epidemiologic heterogeneity of *Cryptosporidium spp*. in the population. PCR-RFLP analysis demonstrated the existence of at least four species of *Cryptosporidium* in the study population; *C*. *hominis*, *C*. *parvum*, *C*. *felis* and *C*. *meleagridis*. Of these four species, *C*. *hominis* which is almost exclusively a human parasite[1,33]was the most common with a prevalence of 82.7%. The other three are zoonotic species but are commonly associated with human cryptosporidiosis [1]. Previous studies on the prevalence of *Cryptosporidium* species and genotypes infecting children in Kenya also found that *C*. *hominis* was the dominant species, and *C*. *parvum*, *C*. *meleagridis*, and *C*. *muris* were identified in HIV-infected persons [34,35]. More outpatients (54%) than inpatients (46%) were infected with *C*. *hominis*, while more inpatients (63%) than outpatients (37%) were infected with *C*. *parvum*. This may imply that *C*. *parvum* caused more severe disease than *C*. *hominis*. This agrees with findings by Cama et al., (2007), where *C*. *parvum* was seen to be more pathogenic. There was equal number of HIV infected and uninfected children found to have *C*. *parvum*, while more HIV negative children were infected with *C*. *hominis*.

This study involved children living in the urban informal settlement of Nairobi, where they live in overcrowded rooms and belong to low socioeconomic classes with poor hygiene and sanitation, but may have minimal direct contact with animals, hence the few zoonotic species. However, the presence of these few zoonotic species including *C*. *felis*, and *C*. *meleagridis* indicates that animal reservoirs are still important. The distribution of *Cryptosporidium* species and genotypes in a population is an indication of the potential infection source, thus, person-to-person transmission probably played an important role in cryptosporidiosis epidemiology in the children in this study. On the other hand, it is difficult to confirm with certainty that the zoonotic species of *Cryptosporidium* were transmitted directly from animal to child, rather than via contamination of water, food, or hands with animal or human faeces. These findings are consistent with results from previous studies in paediatric populations in Africa and other developing countries, where 79–90% of infections are caused by *C*. *hominis* [5,36,37]. In Malaysia and Kuwait, the same four species were identified, however in their study, *C*. *parvum* was the most frequently detected species followed by *C*. *hominis* [38].

*Cryptosporidium felis* is one of the five most common *Cryptosporidium spp*. that are responsible for human cryptosporidiosis. *C*.*felis* was first described in humans in Kenya by Gatei *et al*., (2006b) in two children, and this is the second study to report *C*. *felis* in children in Kenya.

*C*. *meleagridis*, one of the zoonotic species identified in this study has previously been recognized as an important human pathogen in Africa (Kenya), Peru, India and Thailand [39–41]. *C*. *muris* and *C*. *canis* identified in an earlier study by [35] were not among the species isolated in this study. However, an earlier report of possible asymptomatic *C*. *muris* infection in healthy persons [42] and in an immunocompromised patient by[35] suggest that this may be yet another *Cryptosporidium* species with a zoonotic potential.

Given that *C*. *parvum* and *C*. *hominis* constituted 88% of the *Cryptosporidium* infections in the paediatric population studied here, a further evaluation of the genetic variation within each of these two species was carried out using partial *gp40/15* or *GP60*subtyping tool, which is currently, the most common locus for identifying *Cryptosporidium* subtype families and subtypes [28,43]. Understanding the subtypes of *C*. *hominis* and *C*. *parvum* may provide clues into the mechanisms of transmission and infection of these organisms and lay foundations for effective prevention and treatment strategies [21]. The *GP60* analysis of the analysed isolates in this study revealed the high genetic variation of *GP60* subtype families and subtypes in the study population, in which 5 different subtype families and 21 different subtypes within *C*. *hominis* were identified. These included subtype families Ia, Ib, Id, Ie and If, and each had different subtypes. Similar subtype families were reported in children in Uganda, except that they did not detect any subtype family If [44]. In S. Africa and Sweden, the same range of *C*.*hominis* subtype families have been recently reported [45–47].

Four different subtypes within subtype family Ia were reported. A study in Indian urban children identified subtype family Ia to be the most common [48]. Although less frequently reported than *C*. *hominis* Ib, subtype *C*. *hominis* Ia is more genetically diverse at the sub-genotypic level [22]. However, only four subtypes were identified in the study population, which included IaA25R5, IaA27R3, IaA30R3 and IaA7R1. While 3 of the subtypes (IaA27R3, IaA30R3, IaA7R1) have been reported in various parts of the world [48,49], subtype IaA25R5 has not been published previously.

Worldwide, IbA9G3 and IbA10G2 are the two common subtypes within the Ib subtype family. IbA9G3, which is one of the subtypes in this study is commonly seen in humans in Malawi, India, and has been isolated in children [1,40] and in baboons (Li *et al*., 2011). Subtype IbA10G2, commonly seen in South Africa, Botswana, and European countries, and isolated in HIV infected in Jamaica, [1,50–52], and known to be responsible for more than half of the waterborne outbreaks of gastroenteritis was not isolated in this study. However subtype IbA9G3R2 which was present in 3 cases has not been reported in other areas before.

One of the striking findings in our study is the predominance of, and high genetic diversity of subtype family Id in the study population; 31(31%) *C*. *hominis* isolates were identified as subtype Id. This is much higher than prevalence reported earlier, as reviewed by Jex and Gasser, (2010). Subtype IdA22 was the most common as also observed previously by Gatei (2006a or b). In the present study, a relatively rare subtype IdA21 that has only been reported from South Africa, Jordan and China [53–55] was detected. Subtype IdA24, isolated in 3 children has previously been reported in Kenyan children [56], in the US, in a waterborne outbreak of gastroenteritis [57] and in Jordan [55]. Within subtype family Id, subtype IdA15G1, also reported in this study, is usually the most commonly reported, [22]. On the other hand, to the best of our knowledge, subtype IdA23G1, which was isolated from an inpatient has not been reported previously. Therefore, the diversity of Id subtype populations might indicate the presence of a unique *C*. *hominis* genotype transmission in Kenya.

Subtype family Ie is presently of low genetic diversity with only 3 subtypes reported. The *C*. *hominis* Ie subtype identified, IeA11G3T3, has been reported in human infections from other developing countries [1], such as Nigeria [58], and the predominance of subtype IeA11G3T3 in our study agrees with findings by Hira *et al*., (2011). This subtype was also previously isolated from HIV infected persons in Jamaica and Kenya [53]. Likewise, most infections with subtype Ie in humans are caused by IeA11G3T3, but our study identified a less common subtype, Ie11G3T3R1.

The 3 subtypes within the If subtype family reported in this study were; IfA19G1, IfA14G1 and IfA12G1. To the best of our knowledge, this is the first report of subtype If in East Africa and second in humans in Africa after it was detected in South Africa by Samra *et al*., 2012. However one of the subtypes IfA12G2, although not found in this study, has recently been reported in baboons in Kenya [59]. The genetic diversity within the subtype family If differs from the subtypes isolated in Bangladesh where all the 11 isolates were identified as IfA13G1, but in agreement with the study in South African children where two of the subtypes in our study; IfA14G1 and IfA12G1were reported [47]. However subtype IfA19G1, reported in this study, has not previously been reported in Africa.

All 19 *C*. *parvum* samples in our study were of the IIc subtype family. The *C*. *parvum* allelic family IIc has been frequently recorded and described almost exclusively in humans [16,38,46]. Our findings are similar to those observed in Nigeria by Molloy [37], and Cama [13],where IIc was the most common subtype family of *C*. *parvum*. Subtype families IIa and IId which are commonly reported in different parts of the world and are zoonotic [38] were not identified in the present study.

Variation within clades of the same subtype family could be attributed to repeat motif difference; however there was lack of distinct clustering based on either HIV status or patient type (inpatient and outpatient). The most dominant *C*. *hominis* subtype isolated from HIV positive patients was Id (n = 7) and Ib (n = 7) with least genotype Ia and If (n = 1 each).

We observed differences in clinical manifestations among subtype families of *C*. *hominis*: morepersons infected with subtype families Ib, Id and Ie were observed to present with fever, abdominal pains, vomiting and acute diarrhoea, whereas infections with subtype family Ia none had acute diarrhoea. This finding is supported by an earlier study that showed that subtype Id was more virulent than other *C*. *hominis* subtype families in Peruvian HIV-positive people [13], but differs with another study that reported that subtype family Ib may be more pathogenic than Ia, Id, and Ie due to its significant association with diarrhoea, nausea, vomiting and general malaise [25]. On the other hand, the Peru study indicated that infections with the Ia and Ie subtype families were more likely asymptomatic [25]. These results demonstrated that different *Cryptosporidium* subtypes and subtype families may be linked to different clinical manifestations. Indeed, further detailed investigations are warranted in order to improve understanding of these associations.

In conclusion, we demonstrate high genetic diversity of *C*. *hominisGP60* subtype families, and children in the study area may have different clinical responses to infections with different *C*. *hominis* subtype families. Considering that *C*. *hominis* was the predominant species in the study population confirms that transmission in children in this area is predominantly anthroponotic.

## Supporting Information

S1 Fig Alignment showing difference in isolates. (PDF)

S1 Table *Cryptosporidium GP60* subtypes consist of a variable number of ‘‘A” (TCA), ‘‘G” (TCG), ‘‘T” (TCT) and ‘‘R” (ACATCA). (PDF)
