# Supplementary material for: Genetic Diversity of Cryptosporidium in Children in an Urban Informal Settlement of Nairobi, Kenya
Source: PLoS One. 2015 Dec 21;10(12):e0142055. doi: 10.1371/journal.pone.0142055 (PMC4687032; doi:10.1371/journal.pone.0142055)
Supplement: S1 Fig — (PDF) [file pone.0142055.s001.pdf]

```
            1        10        20        30        40        50        60
            |        |         |         |         |         |         |
Gp60-Id   TGAAGGATGTTTCTGTTGAGGGCTCATCATCATCATCATCATCATCATCATCATCATCAT
  MB110   ------------------------------------------------------------
   M580   ------------------------------------------------------------
  MB035   --------------ACCTAGGTTGGTCCTTGTTCAGCTAAGTCTCCGTTCTCATTACAAC
  MB124   --------------------------------------AAGTCTCCGTTCTCATT-CAAC

Gp60-Id   CATCATCATCATCATCAACGACCGTCGCACCAGCTTCAAATAAGGCAAGA-ACTGGAGAG
  MB110   ------------------------------------------------------------
   M580   ------------CATCAACGGCCGTTGCACCAGCTTCAAAAAAGGCAAGAGACTGGAGAG
  MB035   TTGTAGACACCATCGGTGCTGCCGCTATTTTTGCCGGTATAGAAAGCACT-ATCGTCTGC
  MB124   TTGTAGACACCATCGGTGCTGCCGCTATTTTTGCCGGTATAGAAAGCACT-ATCGTCTGC

Gp60-Id   GACACAGGACGAAGCGAAGGAAGTCAAGGTTCTGAAGAACACCAAGACGGAGAGGACGAT
  MB110   ------------------------------------------------------------
   M580   GACACAGGACGAAGCGAAGGAAGTCAAGGTTTTGAAGAACACCAAGACGGAGAGGACGAT
  MB035   AACCAAACTGTACTTGTCTCTTTTCGATGTTTCTTCGGCGTTTGGCACAGCCACTTCAAT
  MB124   AACCAAACTGTACTTGTCTCTTTTCGATGTTTCTTCGGCGTTTGGCACAGCCACTTCAAT

Gp60-Id   AGTTCAGATTCTAGTGGAGGCAGTGTAGGAGGCACAGAGAGCGGAAGTGCAGGAGGAAAG
  MB110   ------------------------------------------------------------
   M580   AGTTCAGATTCTAGTGGAGGCAGTGTAGGAGGCACAGAGAGCGGAAGTGCAGGAGGAAAG
  MB035   TCTCTTACCACCATTAAGGGTAAAGGCAAACAAATCGACGGTTGCAGTTTCACTAGTTTC
  MB124   TCTCTTACCACCATTAAGGGTAAAGGCAAACAAATCGACGGTTGCAGTTTCACTAGTTTC

Gp60-Id   AACGAAGAAGATAGTTCAAGTTCTGGAGGTGCTCAGGACGGCAGTGGAGGCACTGCAGAA
  MB110   --------------------------------TCAGGACGGCAGTGGAGGCACTGCAGAA
   M580   AACGAAGAAGATAGTTCAAGTTCTGGAGGTGCTCAGGACGGCAGTGGAGGCACTGCAGAA
  MB035   CTCTGTGAGTGATCTTCTTGATCTTGACTCAACCTGATTGCCAGACTCACCTTTATTTTC
  MB124   CTCTGTGAGTGATCTTCTTGATCTTGACTCAACATGATGGCCAGAATCACCTCTATTATC

Gp60-Id   GG-CG--CTACTCAGTCCGAGGCTACTGCTTCTCAAGGTGCTCCATCTCAAGGTTCTGAC
  MB110   GGGCG--CTAATCAGTCCGAGGGTACTGCTTCTCAAGGTGCTCCATCTCAAGGTTCTGAC
   M580   GG-CG--CTACTCAGTCCGAGGCTACTGCTTCTCAAGGTGCTCCATCTCAAGGTTCTGAC
  MB035   GGTTGGACTACTTGAGCT-AGCAGAGAGAGTGCTGAAATCCTGACCGTCAA--CCTTGAT
  MB124   GGTTGGACTACTTGAGCT-AGCAGAGAGAGTGATGAAATCCTGACCGTCAA--CCTCGAT

Gp60-Id   AAAACTACCGAGTCCACACAAACTACTCCAAAGGAAGAGTGCGGTACTTCGTTTGTAATG
  MB110   AAAACTACCGAGTCCACACAAACTACTCCAAAGGAAGAGTGCGGTACTTCGTTTGTAATG
   M580   AAAACTACCGAGTCCACACAAACTACTCCAAAGGAAGAGTGCGGTACTTCGTTTGTAATG
  MB035   TTTA--ATTGTATTATCACTTTCTTTTTGGAAGGATACATTCTTTACTTCACCAGAGATA
  MB124   GTTA--ATTGTATTATCACTTTCTTTTTGGAAGGAGACATTCTTTACTTCACCAGAGATA

Gp60-Id   TGGTTCGGTGAAGGTACCCCGGTTGCGACCTTGAAGTGTGGTGGTTACACTATCGTCTAT
  MB110   TGGTTCGGTGAAGGTACCCCGGTTGCGACCTTGAAGTGTGGTGGTTACACTATCGTCTAT
   M580   TGGTTCGGTGAAGGTACCCCGGTTGCGACCTTGAAGTGTGGTGGTTACACTATCGTCTAT
  MB035   TATCTTGGTGCGGG-ATTTGCTTGATCCTTTACAGGTGCATAGACGATAGTGTAACCACC
  MB124   TATCATGGTGCGGG-AGGTGCTTGATCCTTTACAGGTGCATAGACGATAGTGTAACCACC

Gp60-Id   GCACCTGTAAAGGATCAAGCAAAT-CCCGCACCAAGATATATCTCTGGTGAAGTAAAGAA
  MB110   GCACCTGTAAAGGATCAAGCAAAT-CCCGCACCAAGATATATCTCTGGTGAAGTAAAGAA
   M580   GCACCTGTAAAGGATCAAGCAAAT-CCCGCACCAAGATATATCTCTGGTGAAGTAAAGAA
  MB035   ACACTTCAAGGTCGCAACCGGGGTACCTTCACCGAACCACATTACAAACGAAGTACCGCA
  MB124   ACACCTCAAGGTCGCAACCGGGGTACCTTCACCGAACCACATTACAAACGAAGTACCGCA

Gp60-Id   TGTATCCTTCCAAAAAGAAAGTGATAATACAAT--TAAAATCAAGGTTGACGGTCAGGAT
  MB110   TGTATCCTTCCAAAAAGAAAGTGATAATACAAT--TAAAATCAAGGTTGACGGTCAGGAT
   M580   TGTATCCTTCCAAAAAGAAAGTGATAATACAAT--TAAAATCAAGGTTGACGGTCAGGAT
  MB035   CTCTTCCTTTGGAGTAGTTTGTGTGGACTCGGTAGTTTTGTCAGAACCTTGAGATGGAGC
  MB124   [illegible]
```

Supplementary material 1: Alignment showing difference in isolates.

```
Gp60-Id   TTCAGCACTCTCTCTGCTAGCTCAAGTAGTCCAACCGAAAATAAAGGTGAGTCTGGCAAT
  MB110   TTCAGCACTCTCTCTGCTAGCTCAAGTAGTCCAACCGAAAATAAAGGTGAGTCTGGCAAT
```
