# Supplementary material for: Genetic Diversity of Cryptosporidium in Children in an Urban Informal Settlement of Nairobi, Kenya
Source: PLoS One. 2015 Dec 21;10(12):e0142055. doi: 10.1371/journal.pone.0142055 (PMC4687032; doi:10.1371/journal.pone.0142055)
Supplement: S1 Table — (PDF) [file pone.0142055.s002.pdf]

carenum	Genotype	Subtypefamily	Subtype	HIV
M0009	C. hominis	Ia	IaA25R5	NEGATIVE
M0016	C. hominis	Id	IdA22	NEGATIVE
M0044	C. hominis	Id	1dA24	NEGATIVE
M0074	C. hominis	Id	IdA19	NEGATIVE
M0082	C. hominis	Ib	IbA9G3	NEGATIVE
M0121	C. hominis	Id	IdA25	NEGATIVE
M0123	C. hominis	Ia	IaA27R3	NEGATIVE
M0142	C. hominis	Id	IdA22	NEGATIVE
M0187	C. hominis	If	IfA19G1	NEGATIVE
M0194	C. hominis	Ie	Ie11G3T3R1	NEGATIVE
M0246	C. parvum	IIc	IIcA5G3R2	NEGATIVE
M0256	C. hominis	Id	IdA21	NEGATIVE
M0273	C. hominis	Ie	IeA11G3T3R1	NEGATIVE
M0278	C. hominis	Ie	IeA11G3T3R1	NEGATIVE
M0384	C. parvum	IIc	IIcA5G3R2	NEGATIVE
M0466	C. hominis	Id	IdA20	NEGATIVE
M0580	C. hominis	Id	IdA17G1	POSITIVE
M0602	C. parvum	IIc	IIcA5G3R2	POSITIVE
M0679	C. hominis	Id	IdA22	NEGATIVE
M0747	C. parvum	IIc	IIcA5G3R2	NEGATIVE
M0819	C. hominis	Ie	IeA11G3T3R1	NEGATIVE
M0954	C. hominis	Id	IdA18	NEGATIVE
M0986	C. parvum	IIc	IIcA5G3R2	POSITIVE
M1051	C. hominis	Ie	Ie11G3T3R1	NEGATIVE
M1108	C. hominis	Ie	IeA11G3T3R1	NEGATIVE
M1136	C. hominis	Ib	IbA9G3	POSITIVE
M1213	C. hominis	Id	IdA22	POSITIVE
M1227	C. hominis	Id	IdA25	NEGATIVE
M1233	C. hominis	Ib	IbA9G3	NEGATIVE
M1241	C. hominis	Ib	IbA9G3	POSITIVE
M1242	C. hominis	Ie	IeA11G3T3R1	POSITIVE
M1265	C. hominis	Ib	IbA9G3	POSITIVE
M1327	C. hominis	Ie	IeA11G3T3R1	NEGATIVE
M1328	C. hominis	Ib	IbA9G3	POSITIVE
M1362	C. hominis	Ib	IbA9G3	NEGATIVE
M1399	C. hominis	Ib	IbA9G3	NEGATIVE
M1414	C. hominis	Ie	IeA11G3T3R1	NEGATIVE
M1425	C. parvum	IIc	IIcA5G3R2	POSITIVE
M1448	C. hominis	Ie	IeA11G3T3	NEGATIVE
M1454	C. hominis	Ib	IbA9G3	NEGATIVE
M1492	C. hominis	Ia	IaA30R3	POSITIVE
M1499	C. hominis	Ib	IbA9G3	NEGATIVE
M1548	C. hominis	Id	IdA24	NEGATIVE
M1570	C. parvum	IIc	IIcA5G3R2	NEGATIVE
M1599	C. hominis	Ib	IbA9G3	NEGATIVE
M1553	C. hominis	Ia	IaA7R1	NEGATIVE
M1567	C. hominis	Id	IdA22	NEGATIVE
M1574	C. hominis	Id	IdA15G1	NEGATIVE
M1578	C. hominis	Ia	IaA7R1	NEGATIVE

M1593	C. hominis	Ie	IeA11G3T3R1	NEGATIVE
M1605	C. hominis	Ib	IbA9G3	NEGATIVE
MB022	C. hominis	Ib	IbA9G3	NEGATIVE
MB026	C. parvum	IIc	IIcA5G3R2	NEGATIVE
MB035	C. hominis	Id	IdA23GI	NEGATIVE
MB056	C. hominis	Ie	IeA11G3T3R1	NEGATIVE
MB069	C. parvum	IIc	IIcA5G3R2	NEGATIVE
MB084	C. parvum	IIc	IIcA5G3R2	UNKNOWN
MB086	C. hominis	If	IfA14G1	NEGATIVE
MB101	C. hominis	Ie	IeA11G3T3R1	POSITIVE
MB107	C. hominis	Ib	IbA9G3	POSITIVE
MB110	C. hominis	Id	IdA15G1	POSITIVE
MB124	C. hominis	Id	IdA22	POSITIVE
MB151	C. parvum	IIc	IIcA5G3R2	NEGATIVE
MB163	C. parvum	IIc	IIcA5G3R2	POSITIVE
MB178	C. hominis	If	IfA12G1	POSITIVE
MB181	C. hominis	Id	IdA22	NEGATIVE
MB192	C. hominis	Id	IdA22	NEGATIVE
MB214	C. hominis	Ib	IbA9G3R2	NEGATIVE
MB236	C. hominis	Ie	IeA11G3T3R1	NEGATIVE
MB241	C. hominis	Ib	IbA9G3	NEGATIVE
MB254	C. parvum	IIc	IIcA5G3R2	NEGATIVE
MB264	C. hominis	Ie	IeA11G3T3R1	POSITIVE
MB277	C. parvum	IIc	IIcA5G3R2	POSITIVE
MB321	C. parvum	IIc	IIcA5G3R2	POSITIVE
MB322	C. hominis	Ie	IeA11G3T3	NEGATIVE
MB325	C. hominis	Ib	IbA9G3	NEGATIVE
MB330	C. parvum	IIc	IIcA5G3R2	POSITIVE
MB337	C. hominis	Ib	IbA9G3	POSITIVE
MB338	C. parvum	IIc	IIcA5G3R2	POSITIVE
MB350	C. hominis	Id	IdA22	ND
MB355	C. hominis	Ie	IeA11G3T3	ND
MB359	C. hominis	Id	IdA15G1	POSITIVE
MB360	C. hominis	Id	IdA25	POSITIVE
MB360	C. hominis	Id	IdA25	POSITIVE
MB363	C. hominis	Ie	IeA11G3T3R1	NEGATIVE
MB372	C. hominis	Id	IdA22	NEGATIVE
MB389	C. hominis	Ie	IeA11G3T3R1	NEGATIVE
MB397	C. hominis	Id	IdA24	NEGATIVE
MB407	C. hominis	Ie	IeA11G3T3R1	NEGATIVE
MB419	C. hominis	Ib	IbA9G3R2	POSITIVE
MB428	C. hominis	Id	IdA22	POSITIVE
MB435	C. hominis	Ie	IeA11G3T3	POSITIVE
MB441	C. parvum	IIc	IIcA5G3R2	NEGATIVE
MB446	C. hominis	Id	IdA22	POSITIVE
MB456	C. hominis	Id	IdA22	ND
MB490	C. hominis	Ib	IbA9G3R2	POSITIVE
MB493	C. hominis	Id	IdA25	POSITIVE
MB494	C. hominis	Ie	IeA11G3T3R1	NEGATIVE
MB496	C. hominis	Ie	IeA11G3T3R1	POSITIVE

MB501	C. parvum	IIc	IIcA5G3R2	POSITIVE
MB516	C. hominis	Id	IdA22	NEGATIVE

Patienttype
Outpatient
Outpatient
Outpatient
Outpatient
Outpatient
Outpatient
Outpatient
Outpatient
Outpatient
Outpatient
Outpatient
Inpatient
Outpatient
Outpatient
Outpatient
Outpatient
Outpatient
Outpatient
Outpatient
Outpatient
Outpatient
Outpatient
Outpatient
Outpatient
Outpatient
Outpatient
Outpatient
Outpatient
Outpatient
Outpatient
Outpatient
Outpatient
Outpatient
Outpatient
Outpatient
Outpatient
Outpatient
Outpatient
Outpatient
Outpatient
Outpatient
Outpatient
Outpatient
Outpatient
Outpatient
Outpatient
Outpatient
Outpatient
Outpatient

Outpatient
Outpatient
Inpatient
Inpatient
Inpatient
Inpatient
Inpatient
Inpatient
Inpatient
Inpatient
Inpatient
Inpatient
Inpatient
Inpatient
Inpatient
Inpatient
Inpatient
Inpatient
Inpatient
Inpatient
Inpatient
Inpatient
Inpatient
Inpatient
Inpatient
Inpatient
Inpatient
Inpatient
Inpatient
Inpatient
Inpatient
Inpatient
Inpatient
inpatient
Inpatient
Inpatient
Inpatient
Inpatient
Inpatient
Inpatient
Inpatient
Inpatient
Inpatient
Inpatient
Inpatient
Inpatient
Inpatient
Inpatient
Inpatient
Inpatient

Inpatient
Inpatient
